# Diquat Determines a Deregulation of lncRNA and mRNA Expression in the Liver of Postweaned Piglets

**DOI:** 10.1155/2019/9148535

**Published:** 2019-05-12

**Authors:** Jin Wang, Zhi-xin Li, Dan-dan Yang, Pei-qi Liu, Zhi-qiang Wang, Yong-qing Zeng, Wei Chen

**Affiliations:** Shandong Provincial Key Laboratory of Animal Biotechnology and Disease Control and Prevention, College of Animal Science and Technology, Shandong Agricultural University, No. 61 Daizong Street, Tai'an City, Shandong Province 271018, China

## Abstract

Oxidative stress is detrimental to animals and can depress the growth performance and regulate the gene expression of animals. However, it remains unclear how oxidative stress regulates the expression of long noncoding RNAs (lncRNAs) and mRNAs. Therefore, the purpose of this article was to explore the profiles of lncRNAs and mRNAs in the liver of piglets under oxidative stress. Here, we constructed a piglet oxidative stress model induced by diquat and evaluated the effects of oxidative stress on the growth performance and antioxidant enzyme activity of piglets. We also used RNA-Seq to examine the global expression of lncRNAs and mRNAs in piglets under oxidative stress. The targets of lncRNAs and mRNAs were enriched in gene ontology (GO) terms and signaling pathways. The results show that the growth performance and activities of antioxidant enzymes were decreased in piglets under oxidative stress. Moreover, eight lncRNAs (6 upregulated and 2 downregulated) and 30 mRNAs (8 upregulated and 22 downregulated) were differentially expressed in the oxidative stress group of piglets compared to the negative control group. According to biological processes in enriched GO terms, the oxoacid metabolic process, intramolecular oxidoreductase activity, and oxidation-reduction process play important roles in oxidative stress. Pathway analysis showed that the signaling pathways involved in insulin and glucose metabolism had a close relationship with oxidative stress. Further *in vitro* experiments showed that the expression of the upregulated gene *GNMT* was significantly increased in primary porcine hepatocytes after diquat stimulation. In contrast, the level of the downregulated gene *GCK* was significantly decreased at 12 h in primary porcine hepatocytes after diquat stimulation. Our results expand our knowledge of the lncRNAs and mRNAs transcribed in the livers of piglets under oxidative stress and provide a basis for future research on the molecular mechanisms mediating oxidative stress and tissue damage.

## 1. Introduction

In the process of metabolism, organisms can produce many kinds of reactive oxygen species (ROS). These ROS free radicals are maintained at certain physiological steady-state levels, and excessive ROS are generally eliminated by the antioxidant defense system, which includes antioxidant enzymes (superoxide dismutase (SOD) and glutathione peroxidase (GPx)) and nonenzymatic antioxidants (for example, glutathione, Se, vitamin E, and vitamin C) [1]. Therefore, the antioxidant system can protect tissues and cells from ROS damage. When the level of ROS produced by cells is higher than the antioxidant defense ability of cells, the redox state is unbalanced. Excessive ROS in tissues or cells will induce oxidative stress and lead to oxidative damage, such as tissue damage and the oxidation of proteins, lipids, and nucleic acids [2, 3]. In livestock production, numerous factors, such as environmental factors, oxidized diets, and weaning, can induce oxidative stress to damage cellular antioxidant defenses [4, 5]. Oxidative stress can result in suboptimal health conditions of livestock and a reduction in production efficiency and can also cause serious economic losses in animal husbandry systems.

Cells maintain homeostasis through transcription and posttranscriptional regulation that cause changes in gene expression [6, 7]. Long noncoding RNAs (lncRNAs), as important transcripts, play key roles in posttranscriptional mechanisms by regulating RNA stability and translation [8]. lncRNAs are a class of mRNA-like transcripts that are longer than 200 nucleotides (nt) and have no protein-coding potential [9–11]. lncRNAs are involved in numerous biological processes, including cell development and differentiation, immune responses, the cell cycle, and apoptosis, by regulating gene expression [12]. Recent evidence indicates that lncRNAs participate in the response to diverse stressful stimuli [13, 14]. Giannakakis et al. indicated that thousands of lncRNA transcripts were transiently induced by oxidative stress and linked with polysomes, a complex of the mRNA molecule and two or more ribosomes that act to form polypeptide chains during active translation [15]. However, very few oxidative stress-induced lncRNAs have been subjected to functional analysis.

The domestic pig (*Sus scrofa*) is an economically important farm animal worldwide. Pigs, especially weaned piglets on farms, suffer from various challenges, such as weaning, changes in the nutritional source, gastrointestinal disorders, infections, and diarrhea, and these challenges might produce excessive amounts of ROS and induce oxidative stress [4, 16], which may result in the growth retardation of piglets. Diquat (DQ) is a commercially available herbicide and is used extensively in China. It induces animal oxidative stress [5]. Previous research indicated that an ideal piglet model, which is useful for understanding the effects of oxidative stress, could be constructed by intraperitoneal injection of DQ [17, 18]. However, little is known about the effect of oxidative stress on genome-wide lncRNA expression in piglets. Therefore, in the present study, the growth performance of piglets was investigated, and the livers were collected to screen the differentially expressed lncRNAs and mRNAs and pathways using high-throughput sequencing. Furthermore, the key lncRNAs and genes were verified by real-time quantitative polymerase chain reaction (qRT-PCR) to explore the toxicity mechanism of oxidative damage induced by DQ. These results may provide information necessary to propose a new genetic strategy for oxidative stress resistance.

## 2. Materials and Methods

All experimental procedures were conducted in accordance with the protocol approved by the Animal Care and Use Committee of Shandong Agricultural University.

### 2.1. Animals, Housing, and Experimental Design

Landrace piglets weaned at 21 days were collected from 6 litters, and two male pigs were chosen from each litter. The two pigs were divided into an oxidative stress group and a negative control group. After 3 days of adaptation, piglets with an average initial body weight of 5.76 ± 0.22 kg were given free access to water and commercial feed and housed individually in wire cages in constant-temperature (25-27°C) animal rooms with a 12 h light-dark cycle.

At the beginning of the experiment, the oxidative stress group piglets received an intraperitoneal injection of DQ (Sigma-Aldrich, Saint Louis, USA) at 10 mg/kg of body weight, and the negative control group was injected with the same volume of isotonic saline. One piglet in the oxidative stress group died on the 3^rd^ day. The trial lasted for 7 days, and then there were six piglets in the negative control group and five piglets in the oxidative stress group.

### 2.2. Rectal Temperature Measurement and Sample Collection

The rectal temperature of all the pigs was measured at 8:00 am on days 1, 3, 5, and 7. The body weight was also measured at the end of the experiment, and the average daily gain (ADG) was calculated.

All pigs were anesthetized with an intravenous injection of phenobarbital (0.25 mg/kg body weight) and slaughtered by exsanguinations according to protocols approved by the Shandong Agricultural University Animal Care and Use Committee. Fresh liver samples at the same location were collected and divided into two subsamples: one subsample was immediately frozen in liquid nitrogen and then stored at -80°C for extraction of total RNA, and the other subsample was kept in 10% formalin for H&E staining.

### 2.3. Measurement of the Oxidative Stress Index

Liver tissue (200 mg) was homogenized in a tissue lyser (ULTRA-TURRAX® T18 basic, IKA, Germany) for 2 min and then centrifuged for 15 min at 15,000 g at 4°C to remove the cellular debris; the supernatant was collected for oxidative stress index analysis. Total antioxidant capacity (T-AOC), superoxide dismutase (SOD) activity, glutathione peroxidase (GSH-Px) activity, and malondialdehyde (MDA) content in the liver were measured using spectrophotometric kits in accordance with the manufacturer's instructions (Nanjing Jiancheng Biotechnology Institute, Nanjing, China).

### 2.4. Liver Histological Examination

Liver tissue was fixed in 10% formalin for 24 h, followed by embedding in paraffin wax and then cut into 5 *μ*m sections (Leica RM2135, Germany). After 3 h at 60°C, the slides were deparaffinized with xylene, rehydrated with a decreasing series of ethanol, and stained with hematoxylin and eosin (HE) according to standard histological protocols. Slides were photographed with an optical microscope (Olympus BX53).

### 2.5. Library Preparation for lncRNA Sequencing and Data Analysis

According to the rectal temperature, antioxidant enzyme activity, and HE staining of the liver in the oxidative stress piglets, three piglets with the most severe clinical manifestations were chosen from the oxidative stress group. At the same time, the three full sibs of the chosen piglets were also collected from the negative control group. Therefore, six piglets (three piglets in each group) were chosen for the lncRNA sequencing analysis. Total RNA was extracted from liver tissue using a total RNA extractor (Sangon, Shanghai, China) according to the manufacturer's instructions. The quantity and quality of isolated total RNAs were estimated by running on a 1% agarose gel and using a Qubit 2.0 Fluorometer (Invitrogen, Carlsbad, CA, USA). Then, the rRNA was removed using the Ribo-off rRNA Depletion Kit (Vazyme Biotech, Nanjing, China). The lncRNA libraries were generated using the VAHTS™ stranded mRNA-seq library prep kit for Illumina® (Vazyme Biotech, Nanjing, China). The libraries were sequenced using an Illumina HiSeq X Ten platform, and 150 bp paired-end reads were generated.

Sequencing reads or raw reads were trimmed using Trimmomatic (version 0.36) [19] to remove the adaptor sequences, unknown nucleotides larger than 5%, or Q20 < 20%, followed by FastQC (version 0.11.2) quality control checks. Trimmed reads used for downstream analyses had a quality score of Q30. All clean reads were aligned to the reference genome of *Sus scrofa* (*Sscrofa* 10.2; ftp://ftp.ensembl.org/pub/release-75/fasta/sus_scrofa/) using the HISAT2 (version 2.1.0) software [20].

The transcript expression levels were calculated using the transcript per million (TPM) values generated by the StringTie (version 1.3.3b) software [21], and then the expression data was normalized using the R package RUVSeq (version 1.17.1) [22]. To obtain high-quality lncRNAs, transcripts with lengths greater than 200 nt were used for the prediction. The coding potential of these transcripts was predicted using CPC2 (version 2.0) [23], CNCI (version 2.0) [24], Pfam [25], and PLEK (version 1.2) [26]. The selected transcripts without coding potential from these software analysis results were considered lncRNAs. The lncRNAs and mRNAs (TPM > 0.1) that were detected commonly in all six samples were assigned as co-genes. The differentially expressed (DE) lncRNAs and mRNAs were analyzed using the R package DESeq [27]. The lncRNAs and mRNAs with fold changes (log2 ratio) ≥ 2 and *q*value significance scores < 0.05 were regarded as DE lncRNAs and mRNAs.

All the DE genes and the predicted target genes of DE lncRNAs were submitted to the databases of gene ontology (GO) and Kyoto Encyclopedia of Genes and Genomes (KEGG) for enrichment analysis.

### 2.6. Isolation of Primary Porcine Hepatocytes and Oxidative Stress Treatment

The five three-day-old pig livers were perfused, and hepatocytes were isolated and purified using the two-step procedure described by Fang et al. [28]. The isolated hepatocytes were resuspended in RPMI 1640 medium supplemented with 10% fetal bovine serum, and the hepatocytes (5 × 10^6^ cells/mL) were treated with 50 *μ*M DQ for 6 and 12 h. Control cells were cultured under the same conditions except without DQ treatment. Total RNA of the primary porcine hepatocytes was extracted using TRIzol (Takara Biotechnology, Dalian, China) according to the manufacturer's instructions. The quantity and quality of the isolated RNA were determined via UV260/280 using a biophotometer (Eppendorf, Hamburg, Germany). The *GNMT* and *GCK* expression levels in primary porcine hepatocytes were determined by qRT-PCR.

### 2.7. Quantitative Real-Time RT-PCR (qPCR)

Eight DE lncRNAs and 13 DE mRNAs (Table S1) were selected to verify the expression changes observed in our RNA-Seq results. Primers of selected lncRNAs and mRNAs were designed with Primer3web software (version 4.1.0) [29] and then synthesized by Sangon Biotech (Shanghai, China). Real-time PCR was performed using the SYBR® Premix Ex Taq^TM^ (Takara, Dalian, China) and a LightCycler® 96 Real-Time PCR System (Roche). Briefly, the amplifications were performed with a 20 *μ*L reaction volume containing 10 *μ*L of 2× SYBR Premix ExTaq, 0.5 *μ*L of each primer, 2 *μ*L of diluted cDNA, and sterile water to the volume to 20 *μ*L. The PCR amplification was carried out as follows to calculate the melting curve: 95°C for 10 s; then 40 cycles of 95°C for 5 s, 58°C for 10 s, and 72°C for 15 s; followed by 1 cycle of 95°C for 1 min, 61°C for 30 s, and 95°C for 30 s. To exclude between-run variations, all samples were amplified in triplicate, and the mean was used for further analysis. A negative control reaction without template was performed for each primer to ensure that RNAs were free of genomic DNA. *GAPDH* and *TOP2B* genes were used as a control for expression normalization. Only primers showing an efficiency between 85 and 115% were used for qRT-PCR. The comparative cycle threshold (CT) method (2^−ΔΔCT^) was used to quantitate mRNA expression [30].

### 2.8. Statistical Analyses

Data were analyzed by using the statistical R package (http://www.r-project.org/), and the results are presented as the mean ± standard error of the mean (SEM). The statistical significance of differences between two groups was compared with an unpaired two-tailed Student *t*-test. The differences were considered statistically significant when ^∗^ *P* < 0.05, ^∗∗^ *P* < 0.01, and ^∗∗∗^ *P* < 0.001.

## 3. Results

### 3.1. Growth Performance

**Figure 1:**
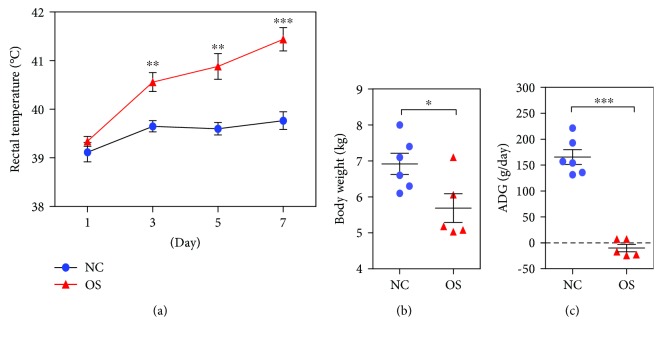
The effects of DQ on piglet performance between the oxidative stress group (*n* = 5) and the negative control group (*n* = 6). (a) The rectal temperature of piglets changes at days 1, 3, 5, and 7. (b) The last body weight at the end of the experiment. (c) The effect of DQ on average daily gain (ADG). OS: oxidative stress group; NC: negative control group. ^∗^ *P* < 0.05, ^∗∗^ *P* < 0.01, and ^∗∗∗^ *P* < 0.001.

The average rectal temperature of the oxidative stress group was significantly higher than that of the negative control group on days 3, 5, and 7 (Figure 1(a)). However, the injection of DQ significantly decreased the average body weight (Figure 1(b)), and the average daily gain (ADG) of the oxidative stress group was significantly lower than that of the negative control group (Figure 1(c)).

### 3.2. Activities of Antioxidant Enzymes and MDA Concentrations in the Liver

**Figure 2:**
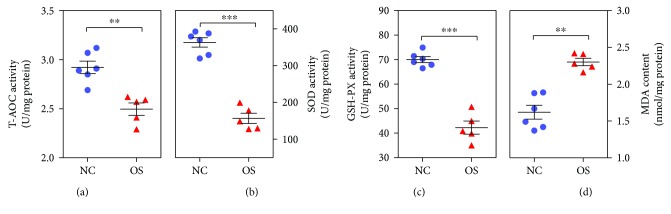
The effects of DQ on total antioxidant capacity (T-AOC) activity (a), superoxide dismutase (SOD) activity (b), glutathione peroxidase (GSH-Px) activity (c), and malondialdehyde (MDA) content (d) between oxidative stress (*n* = 5) and negative control (*n* = 6) groups. OS: oxidative stress group; NC: negative control group. ^∗^ *P* < 0.05, ^∗∗^ *P* < 0.01, ^∗∗∗^ *P* < 0.001.

It was observed that animals injected with DQ at 10 mg/kg of body weight presented significantly lower activity of T-AOC (Figure 2(a)), SOD (Figure 2(b)), and GSH-PX (Figure 2(c)) than the negative control group pigs. However, the MDA contents in oxidative stress group piglets were significantly higher than those in the negative control piglets (Figure 2(d)).

### 3.3. Effects of DQ on Liver Morphological Structure

**Figure 3:**
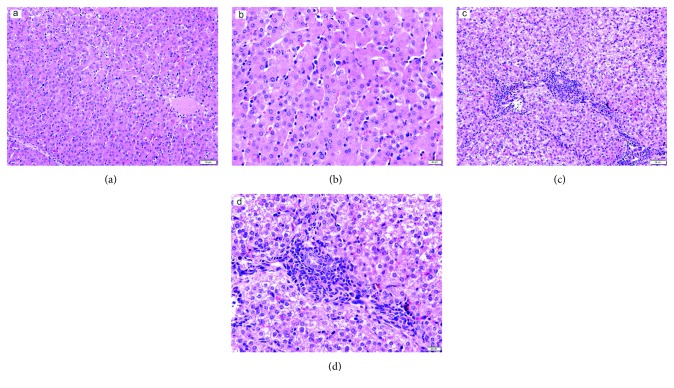
Histological evaluation of liver tissues after exposure to diquat: (a) negative control group (HE, ×200), (b) negative control group (HE, ×400), (c) oxidative stress group (HE, ×200), and (d) oxidative stress group (HE, ×400).

The liver is the major target organ of DQ [31]; therefore, the injection of DQ significantly influenced the liver morphological structure. Compared to the negative control group (Figures 3(a) and 3(b)), the liver of oxidative stress group piglets was in an oxidative stress state, and H&E staining results showed extensive empty package degeneration, hepatocyte disintegration, seriously disordered hepatic cords, hepatic sinusoidal congestion, and the interstitial infiltration of red blood cells and inflammatory cells. The liver was in an inflammatory state, accompanied by hepatocyte vacuolar degeneration in the DQ group. The hepatocytes were swollen, granular, and partially necrotic (Figures 3(c) and 3(d)).

### 3.4. Overview of the Sequencing Data

To understand which DQ-sensitive RNAs were involved in the liver, RNA-Seq analysis was performed in the oxidative stress group and negative control group (three samples per group). After filtering the 3′ adaptor sequence and discarding low-quality reads, a high percentage of the reads was mapped to the pig reference genome, ranging from 82.52 to 84.48%. Among the mapped reads, 69.09 to 74.65% were mapped uniquely to the pig reference genome. All six samples had nearly 95% of reads equal to or exceeding Q30 (Table 1).

### 3.5. Identification and Analysis of DE lncRNAs and mRNAs

Compared to the negative control group, eight lncRNAs were differentially expressed in the DQ-induced liver samples, in which six lncRNAs were upregulated and the other two lncRNAs were downregulated (Table S2). Additionally, a total of 30 mRNAs were differentially expressed in the liver of the DQ-induced oxidative stress group: eight genes (*PCK1*, *HMGCS2*, *VCL*, *HAMP*, *APOA4*, *GNMT*, *ENSSSCG00000015054*, and *ILF3*) were upregulated, and 22 genes were downregulated in the oxidative stress group (Table S2).

### 3.6. GO and KEGG Pathway Enrichment Analysis of DE lncRNAs and mRNAs

**Figure 4:**
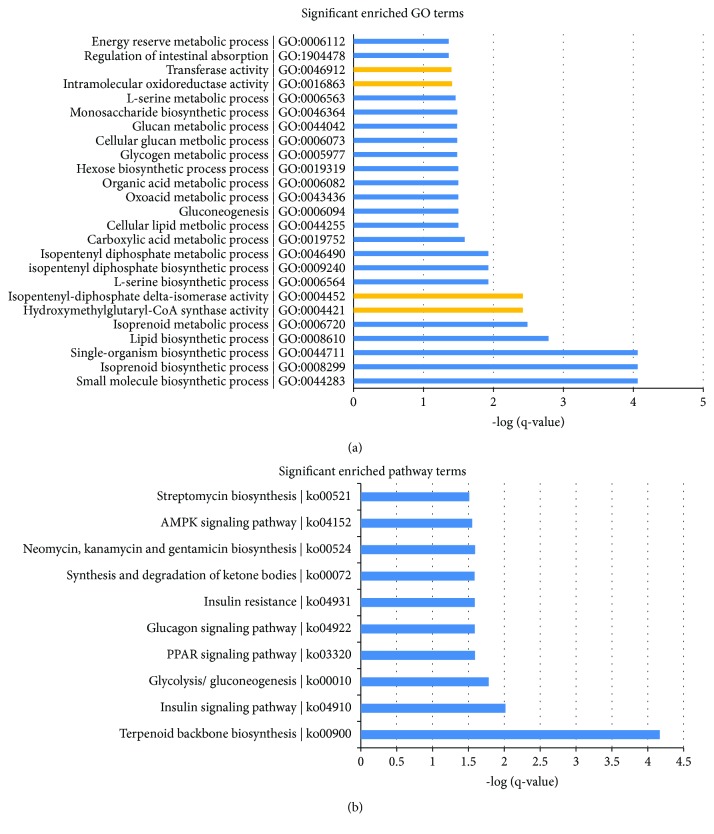
GO (a) and KEGG pathway (b) enrichment analysis of DE lncRNAs and mRNAs.

To further understand the functions of these DE genes, we performed GO enrichment analysis, which produced terms arranged and displayed according to biological processes, molecular functions, and cellular components (Figure 4(a)) to identify the gene expression changes associated with DQ treatment. The significantly enriched categories of our gene set were small-molecule biosynthetic process, isoprenoid biosynthetic process, extracellular matrix structural constituent, hydroxymethylglutaryl-CoA synthase activity, and isopentenyl-diphosphate delta-isomerase activity. In addition to these terms, we were also interested to find that the oxidation-reduction process played an important role in the biological processes involved in these altered genes. The biological process of the oxidation-reduction process serves to protect the body from oxidative damage, maintaining a dynamic balance.

We determined the KEGG pathways related to the differentially expressed genes in the liver. The results, as shown in Figure 4(b), indicated that DQ caused a change in 10 signaling pathways involved in the sensitive-expressed RNAs in piglets. Among these, the insulin signaling pathway; glycolysis/gluconeogenesis; the PPAR signaling pathway; the glucagon signaling pathway; insulin resistance; the synthesis and degradation of ketone bodies; neomycin, kanamycin, and gentamicin biosynthesis; and the AMPK signaling pathway are oxidative stress-relevant pathways.

### 3.7. DE Genes Associated with Oxidative Stress

Among the DE mRNAs, we found that *GNMT*, protein phosphatase 1 regulatory subunit 3C (*PPP1R3C*), *SCD*, *APOA4*, *GCK*, and *ENSSSCG00000027643* were enriched under the oxidation-reduction process term. *GNMT* and *APOA4* were upregulated genes in the DQ-induced oxidative stress group, and the other genes were downregulated. Other oxidative stress-related genes, including *CHAC1*, *HMGCS1*, *PSAT1*, and *PSPH*, were all differentially expressed between the two groups. *ILF3*, as an immune-related gene, was upregulated in the oxidative stress group and was also associated with oxidative stress.

### 3.8. qPCR Validation of the Gene Expression Data from RNA-Seq

**Figure 5:**
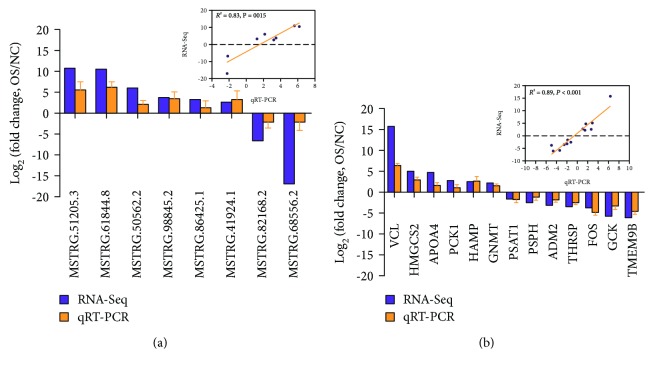
Validation of eight DE lncRNAs and 13 DE mRNAs identified from RNA-Seq results using qPCR. There was a significant correlation between transcript fold-change values determined by RNA-Seq and qPCR, and *R* ^2^ = 0.83 for DE lncRNAs (a) and 0.89 for DE mRNAs (b).

To verify the gene and lncRNA expression data by RNA-Seq, qPCR was performed for eight DE lncRNAs and 13 DE mRNAs for oxidative stress, including six upregulated genes and seven downregulated genes in oxidative stress group piglets (*n* = 5) compared to negative control group piglets (*n* = 6). The results showed that all tested lncRNAs and mRNAs were consistent between RT-qPCR and sequencing analysis, and there was a significant correlation (*R* ^2^ = 0.83 for DE lncRNAs and 0.89 for DE genes; Figures 5(a) and 5(b), respectively). Besides, the expression levels of the DE lncRNAs and mRNAs were also performed in primary porcine hepatocytes after DQ stimulation 12 h, and the results showed that there was the same expression tend (Figure S1). These results suggest that our estimation of abundance was accurate.

### 3.9. Oxidation-Reduction Process-Related Gene Expression

**Figure 6:**
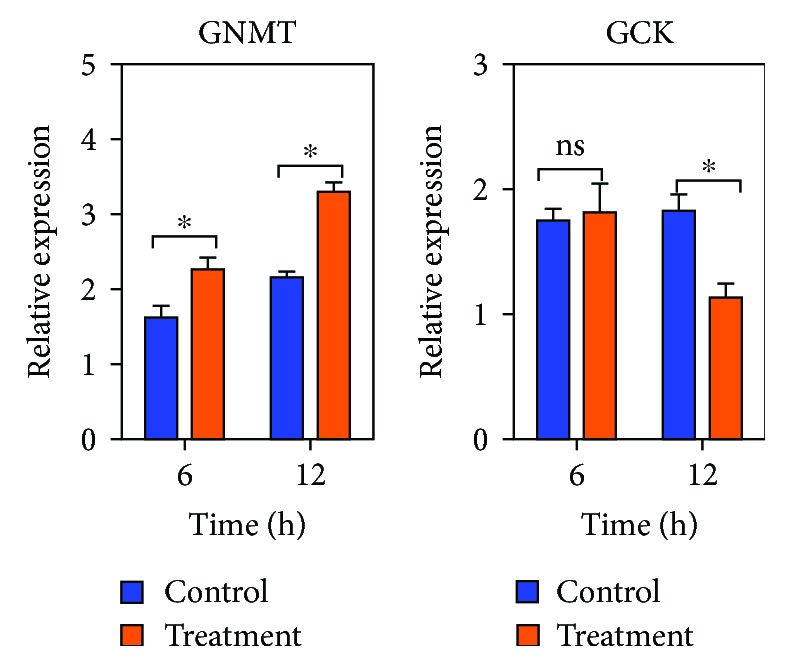
The expression of *GNMT* and *GCK* in primary porcine hepatocytes (*n* = 5). Treatment: the isolated hepatocytes were treated with 50 *μ*M DQ for 6 and 12 h. Control: the control cells were cultured under the same conditions except without DQ treatment. ^∗^ *P* < 0.05; ns: not significant.

Because the GO term oxidation-reduction process is very important, we selected GNMT (upregulated) and GCK (downregulated) from the GO term and further studied their expression in primary porcine hepatocytes after DQ stimulation. The expression level of the *GNMT* gene after 6 (*P* = 0.037) and 12 h (*P* = 0.021) stimulation with DQ was significantly higher than that in the control group (Figure 6(a)). After stimulation with DQ, the *GCK* mRNA level was significantly decreased in the treatment group at 12 h (*P* < 0.05); however, there was no difference between the control group and treatment group at 6 h (Figure 6(b)). These results suggest that the DQ treatment had a significant effect on oxidation-reduction process signaling.

## 4. Discussion

DQ, as a hepatotoxic bipyridyl herbicide [32, 33], is widely applied in the field and is used to construct animal oxidative stress models *in vivo* [17]. Previous research has reported that DQ-induced oxidative stress decreases porcine growth performance and nutritional metabolism ability [4, 34, 35]. In the present study, we also found that the ADG was decreased in oxidative stress group pigs, which disrupted the redox state. The activity of T-AOC, SOD, and GSH-Px in the liver was decreased after DQ injection in piglets. Oxidative stress results from the failure of antioxidant enzymes to eliminate free radicals [36]. Thus, the ROS produced in mitochondria cannot decompose superoxide anions (O_2_^·−^) to hydrogen peroxide (H_2_O_2_) [37]; therefore, O_2_^·−^ causes cellular injury and liver damage.

Although many studies have shown that DQ results in damage to the liver and the liver is the major target of DQ, the genetic and regulatory mechanisms resulting in oxidative stress remain unclear. As a kind of high-throughput detection technology, RNA-Seq has been widely used in many fields. To further analyze the molecular mechanism of damage induced by DQ, the livers of pigs were collected to screen the DE mRNAs, lncRNAs, and signaling pathways using RNA-Seq. Overall, there were significant differences in liver mRNA and lncRNA expression between the oxidative stress and NC groups. There were 30 DE mRNAs and 8 DE lncRNAs between the two groups.

lncRNAs have been identified in numerous porcine tissues, such as the skeletal muscles [38], lung [39], and spleen tissues [40], and play an important role in major mechanisms of gene expression, regulation, and cellular development. Despite the biological functions of lncRNAs, it is not yet clear whether lncRNAs are involved in the regulation of oxidative stress in the liver of piglets. In our present study, eight DE lncRNAs were identified, revealing the lncRNAs participating in the regulation of oxidative stress induced by DQ. In addition, the GO analysis also showed that the targets of lncRNAs were mainly enriched in lipoprotein oxidation. Our results provide a molecular basis for studying the function of lncRNAs in oxidative stress induced by DQ.

GNMT is the most abundant methyltransferase in the liver and plays an important role in *S*-adenosylmethionine homeostasis as it methylates glycine to form sarcosine [41]. Previous research has reported that *GNMT* regulates genes related to the antioxidation pathways [42, 43]. In the present study, *GNMT* was highly expressed in the oxidative stress group compared with the NC group. Furthermore, we detected *GNMT* expression levels in primary porcine hepatocytes *in vitro*, and the results showed that *GNMT* was also highly expressed in hepatocytes after stimulation with DQ. These findings agree with a previous study showing that *GNMT* may prevent oxidative stress-induced liver damage [44].

Because it can catalyze the conversion of glucose to glucose-6-phosphate, GCK plays an important role in glucose metabolism [45]. It has been reported that *GCK* is essential for the regulation of various glucose-responsive genes in hepatocytes [46]. In our present study, the expression of *GCK* was downregulated in the oxidative stress group and decreased significantly in hepatocytes after 12 h of treatment with DQ. These results suggest that the rate of both glucose uptake and glycogen synthesis in the liver was inhibited; therefore, the ADG of oxidative stress group piglets was decreased.

Mitochondrial 3-hydroxy-3-methylglutaryl-CoA synthase (HMGCS2) delivers lipid-derived energy to liver cells, and suppression of HMGCS2 in the nonalcoholic steatohepatitis livers could indicate impairment of ketogenesis and cholesterol biosynthesis [47]. Additionally, the upregulated gene *HMGCS2* is also correlated with fatty acid oxidation induced by a high-fat diet [48]. Moreover, *ILF3* is an immune-related gene regulating p21 expression in leukemic cells and is associated with dichloroacetate-mediated cytotoxicity [49]. In our present study, these two genes displayed increased expression in oxidative stress group piglets, suggesting that the upregulation of *HMGCS2* and *ILF3* was resistant to oxidative stress in the liver.

## 5. Conclusion

In summary, the results of this study showed that DQ-induced oxidative stress decreased growth performance and led to the differential expression of lncRNAs and mRNAs in the livers of piglets. Eight DE lncRNAs and 30 DE mRNAs were identified, and the target genes of lncRNAs and mRNAs were enriched in the oxidation-reduction process. Moreover, our study also provides a reference for lncRNAs and mRNAs that can be used for biomedical research related to oxidative stress diseases in the future.

## Figures and Tables

**Table 1:** A summary of the sequencing read alignment to the *Sus scrofa* genome.

Sample	NC1		NC2		NC3		OS1		OS2		OS3	
	Count	%	Count	%	Count	%	Count	%	Count	%	Count	%
Total reads	56879106		36876312		31523852		23752128		33136858		34721210	
Total mapped	47350312	83.25	30742830	83.37	26632830	84.48	20008310	84.24	27346751	82.53	28653139	82.52
Multiple mapped	7509799	13.20	4866020	13.20	3100574	9.84	3598810	15.15	2678440	8.08	2916956	8.40
Uniquely mapped	39840513	70.04	25876810	70.17	23532256	74.65	16409500	69.09	24668311	74.44	25736183	74.12
%≥Q30	95.33		95.02		95.00		94.84		95.23		95.28	
GC content (%)	61.98		62.92		62.06		67.01		57.83		57.31	

## Data Availability

The data used to support the findings of this study are available from the corresponding authors upon request.

## Supplementary Materials

Supplementary Materials Supplemental Table S1: primer information of differentially expressed lncRNAs and genes for qPCR. Supplemental Table S2: differentially expressed lncRNAs and mRNAs between the two groups. Supplemental Figure S1: the expression of DE lncRNAs and mRNAs in primary porcine hepatocytes.
