# Supplementary material for: Diquat Determines a Deregulation of lncRNA and mRNA Expression in the Liver of Postweaned Piglets
Source: Oxid Med Cell Longev. 2019 May 12;2019:9148535. doi: 10.1155/2019/9148535 (PMC6535875; doi:10.1155/2019/9148535)
Supplement: Supplementary Materials — Supplemental Table S1: primer information of differentially expressed lncRNAs and genes for qPCR. Supplemental Table S2: differentially expressed lncRNAs and mRNAs between the two groups. Supplemental Figure S1: the expression of DE lncRNAs and mRNAs in primary porcine hepatocytes. [file 9148535.f1.pdf]

Supplementary materials: Table S1 Primers information of differentially expressed lncRNAs and genes

Gene id	MeanTPM (OS)	MeanTPM (NC)	log2FoldChange	p-Value	q-Value	result	forward primer (5'-3')	reverse primer (5'-3')
**lncRNA**								
MSTRG.82168.2	0.383333333	41.726667	-6.76622641	2.83E-12	1.46E-08	down	GTTGGGCCGCAGTTACAG	GGGTCTGGGTCTGAAGCA
MSTRG.68556.2	1.00E-04	12.296667	-16.90790776	2.15E-07	0.000181	down	CGTCAGCTGGAGAACCTCA	GTGTACCACGAACGCCACT
MSTRG.41924.1	227.54	35.966667	2.66138782	1.37E-08	2.12E-05	up	TCACCATCGGAAACTGACAA	AGGACGTTCCCCGTCTAGTT
MSTRG.51205.3	6.32	0.0033333	10.88874325	7.21E-10	1.82E-06	up	GTCGTGGTGGTGATGAAGTG	TCACAGTCACAGGCTTCTGG
MSTRG.86425.1	14.9	1.57	3.246475866	8.21E-06	0.004253	up	CGTCAGAAAGATGTCCAGCA	CTCCTGTGGGGAACACAGTT
MSTRG.98845.2	13.29333333	0.9933333	3.742281174	1.13E-07	0.000104	up	CCTCGCTCAGTAGGTGAAGG	TCAGCATTCCTTGTTTGCAG
MSTRG.61844.8	93.46333333	0.0633333	10.52721976	9.27E-10	1.87E-06	up	GCCATCGAAGGAGCAGTTAG	TGTAGGTGCTGTCCTTGCTG
MSTRG.50562.2	8.783333333	0.14	5.971269824	1.31E-11	4.40E-08	up	TCACCATCGGAAACTGACAA	GGTGGTGAAGATGTGACACG
**gene**								
VCL	5.806667	0.0001	15.82542	2.88E-12	1.46E-08	up	CAGACCTGCTCCTCACCTTC	TGGTGAGTCAGTTCCTGCTG
HMGCS2	228.4433	6.623333	5.108135	5.04E-12	2.04E-08	up	CAGGACTCAGGCAACACTGA	GACAGCGATGTCTCCACAGA
APOA4	768.3033	28.11667	4.772179	2.04E-05	0.008414	up	ACACCTACACGGAGGACCTG	CTGAACCTGGGTGTTGACCT
PCK1	3419.947	516.9067	2.725998	1.53E-05	0.006885	up	CTTGGGAAGAAGTGCTTTGC	AATTTCATCCAGGCGATGTC
HAMP	150.3433	24.58667	2.612313	2.36E-05	0.009361	up	CCCAGACAAGACAGCTCACA	GCTCTACGTCTTGCAGCACA
GNMT	508.31	108.67	2.225755	9.81E-06	0.004832	up	GCAGCTGTACATCGGAGACA	GGCATCCACACTTGTCACAC
PSAT1	35.33	109.0667	-1.62624	1.50E-05	0.006885	down	CGCTCGGTATTGTTGGAGAT	CCACCTCCTTGCACAAAAAT
PSPH	3.84	22.56	-2.55459	1.05E-08	1.76E-05	down	GTTTGCCAACAGGCTGAAAT	GTGGCTCCATCTCCAATCAT

ADM2	2.093333	19.05333	-3.18617	2.07E-06	0.001347	down	TGCATCAGCCTCCTCTACCT	CTTCTCTGTGGCTGGAGAGC
THRSP	11.84333	137.4167	-3.53641	2.55E-10	7.37E-07	down	AAGTGCTGTTGGGAATGCTT	CTGGCCAGGTAACATCTGGT
FOS	12.66333	177.0867	-3.80573	1.51E-06	0.001089	down	AGAATCCGAAGGGAAAGGAA	CTTCTCCTTCAGCAGGTTGG
GCK	1.076667	60.09333	-5.80256	6.38E-15	1.29E-10	down	CCACTGTCACCCCAGAAGAT	CTTCTGGGTCCTTGATTCCA
TMEM9B	0.35	23.88667	-6.09271	9.45E-08	9.54E-05	down	ACATGATCTGGGTTCCTTGC	CTCAATCTGCCCCATCACTT

Supplementary materials: Table S2: Differentially expressed lncRNAs and mRNAs between the two groups

lncRNA id	MeanTPM (OS)	MeanTPM (NC)	log2FoldChange	pValue	qValue	result		
MSTRG.41924.1	227.54	35.96666667	2.66138782	1.37E-08	2.12E-05	up		
MSTRG.51205.3	6.32	0.003333333	10.88874325	7.21E-10	1.82E-06	up		
MSTRG.86425.1	14.9	1.57	3.246475866	8.21E-06	0.0042528	up		
MSTRG.82168.2	0.383333333	41.72666667	-6.76622641	2.83E-12	1.46E-08	down		
MSTRG.98845.2	13.29333333	0.993333333	3.742281174	1.13E-07	0.0001041	up		
MSTRG.61844.8	93.46333333	0.063333333	10.52721976	9.27E-10	1.87E-06	up		
MSTRG.68556.2	0.0001	12.29666667	-16.90790776	2.15E-07	0.0001811	down		
MSTRG.50562.2	8.783333333	0.14	5.971269824	1.31E-11	4.40E-08	up		
**mRNA id**	**MeanTPM (OS)**	**MeanTPM (NC)**	**log2FoldChange**	**pValue**	**qValue**	**result**	**GeneID**	**GeneName**
ENSSSCT00000016242	12.39333333	141.47	-3.512859974	8.56E-10	1.87E-06	down	ENSSSCG00000024447	-
ENSSSCT00000008222	3419.946667	516.9066667	2.725998112	1.53E-05	0.0068847	up	ENSSSCG00000007507	PCK1
ENSSSCT00000022401	2.093333333	19.05333333	-3.186169452	2.07E-06	0.0013475	down	ENSSSCG00000023749	ADM2
ENSSSCT00000024294	0.213333333	6.01	-4.816183681	7.89E-07	0.0005902	down	ENSSSCG00000027643	-
ENSSSCT00000007357	228.4433333	6.623333333	5.108135064	5.04E-12	2.04E-08	up	ENSSSCG00000006716	HMGCS2
ENSSSCT00000035614	7.556666667	57.35	-2.923971596	2.55E-06	0.0016083	down	ENSSSCG00000010554	SCD
ENSSSCT00000005253	0.74	26.93666667	-5.185902252	4.60E-06	0.0025797	down	ENSSSCG00000004754	CHAC1
ENSSSCT00000011060	0.286666667	7.72	-4.751154783	5.18E-08	6.15E-05	down	ENSSSCG00000010099	-
ENSSSCT00000011290	5.806666667	0.0001	15.8254226	2.88E-12	1.46E-08	up	ENSSSCG00000010313	VCL
ENSSSCT00000016240	11.84333333	137.4166667	-3.5364099	2.55E-10	7.37E-07	down	ENSSSCG00000020745	THRSP
ENSSSCT00000018376	2.65	21.24	-3.002719501	8.48E-08	9.01E-05	down	ENSSSCG00000016872	HMGCS1

ENSSSCT00000002650	12.66333333	177.0866667	-3.805726475	1.51E-06	0.0010891	down	ENSSSCG00000002383	FOS
ENSSSCT00000005824	35.33	109.0666667	-1.626244593	1.50E-05	0.0068847	down	ENSSSCG00000005287	PSAT1
ENSSSCT00000032697	0.0001	6.116666667	-15.90045804	2.81E-13	2.84E-09	down	ENSSSCG00000001391	CCHCR1
ENSSSCT00000018233	1.076666667	60.09333333	-5.80256138	6.38E-15	1.29E-10	down	ENSSSCG00000016751	GCK
ENSSSCT00000025266	20.48	133.3433333	-2.702858077	5.58E-06	0.0030443	down	ENSSSCG00000027363	-
ENSSSCT00000003189	150.3433333	24.58666667	2.612312836	2.36E-05	0.0093605	up	ENSSSCG00000002886	HAMP
ENSSSCT00000016434	768.3033333	28.11666667	4.77217854	2.04E-05	0.008414	up	ENSSSCG00000015068	APOA4
ENSSSCT00000026557	42.2	175.39	-2.05525159	2.31E-05	0.0093164	down	ENSSSCG00000029074	-
ENSSSCT00000015921	0.35	23.88666667	-6.092706811	9.45E-08	9.54E-05	down	ENSSSCG00000014577	TMEM9B
ENSSSCT00000011452	4.686666667	35.05333333	-2.902917548	3.92E-06	0.0022613	down	ENSSSCG00000010464	-
ENSSSCT00000001837	508.31	108.67	2.225754896	9.81E-06	0.0048321	up	ENSSSCG00000001652	GNMT
ENSSSCT00000008495	3.84	22.56	-2.554588852	1.05E-08	1.76E-05	down	ENSSSCG00000007748	PSPH
ENSSSCT00000016420	116.62	9.05	3.687753625	9.14E-06	0.0046159	up	ENSSSCG00000015054	-
ENSSSCT00000011546	7.536666667	50.28666667	-2.738177434	6.08E-06	0.0032298	down	ENSSSCG00000010554	SCD
ENSSSCT00000001573	0.98	7.32	-2.900989994	5.89E-08	6.61E-05	down	ENSSSCG00000023611	-
ENSSSCT00000034166	17.17	137.1133333	-2.997406926	3.46E-06	0.0021167	down	ENSSSCG00000007978	-
ENSSSCT00000024912	17.13	113.18	-2.724021986	1.96E-06	0.0013213	down	ENSSSCG00000029066	-
ENSSSCT00000034746	14.22	0.12	6.888743249	6.04E-09	1.11E-05	up	ENSSSCG00000013638	ILF3
ENSSSCT00000032157	37.00666667	165.7066667	-2.162774548	3.89E-06	0.0022613	down	ENSSSCG00000027593	-

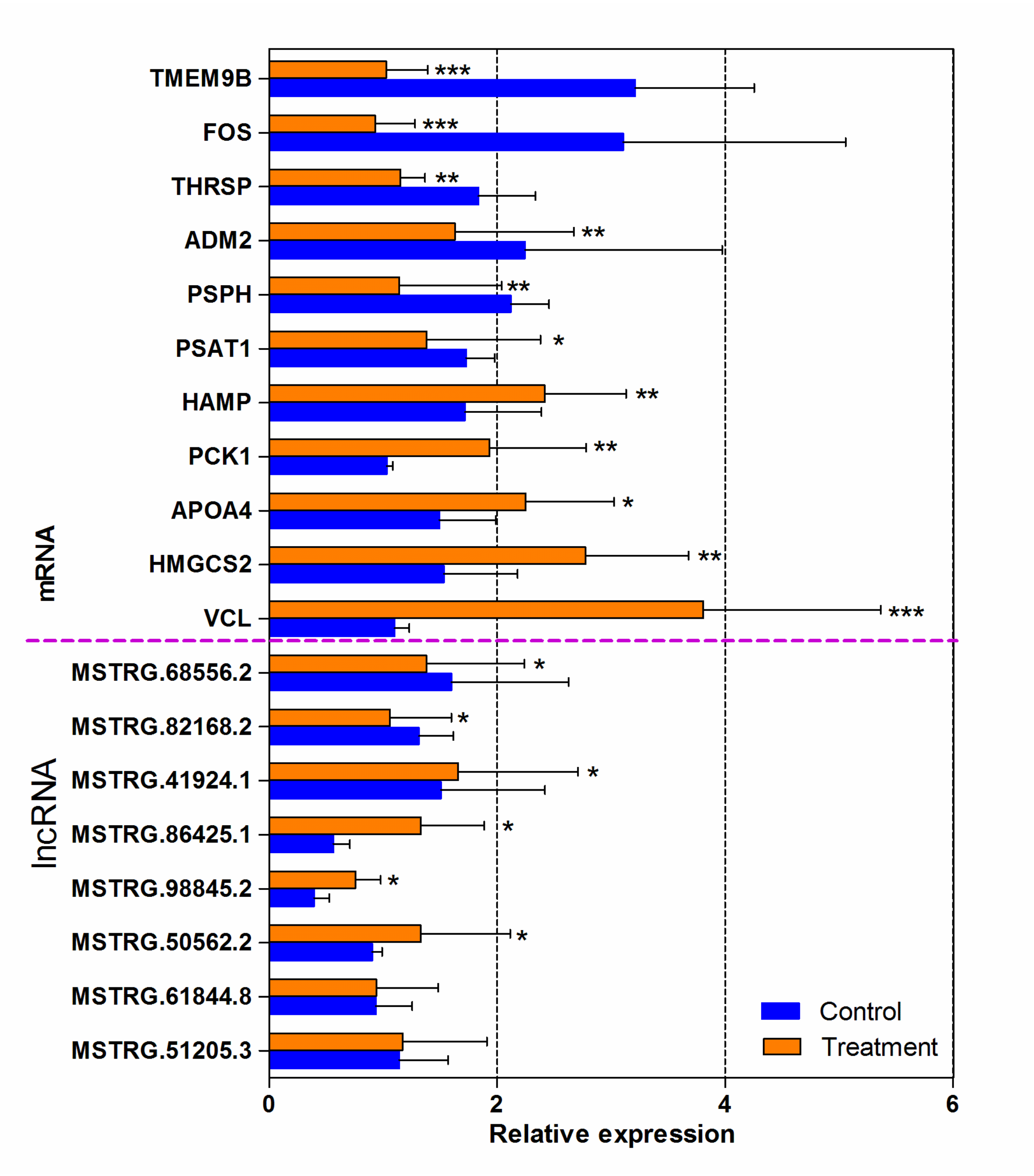

Supplemental Figure S1: The expression of DE lncRNAs and mRNAs in primary porcine hepatocytes. Treatment: the isolated hepatocytes were treated with 50 µM DQ for 12 hours. Control: the control cells were cultured under the same conditions except without DQ treatment. * $P < 0.05$, ** $P < 0.01$, *** $P < 0.001$.
